# A multiplicative behavioral model of DNA replication initiation in cells

**DOI:** 10.1515/biol-2025-1229

**Published:** 2025-12-30

**Authors:** Tahir Rahman

**Affiliations:** School of Medicine, Alvin J. Siteman Cancer Center, Washington University in St. Louis, St. Louis, MO, USA

**Keywords:** DNA replication initiation; cell-cycle checkpoints; origin licensing; chromatin accessibility; threshold model, ARCH × Φ model

## Abstract

DNA replication is a precisely timed cellular decision rather than a continuous biochemical process. Despite extensive mechanistic detail, no unified framework quantitatively explains how structural, metabolic, chromatin, and phase-dependent factors converge to initiate replication. Here, we introduce the ARCH × Φ model, which defines replication onset as a multiplicative threshold event integrating structural, metabolic, chromatin, and phase-control domains. Derived from the recently formalized ARCH behavioral framework, the model expresses replication initiation as *R* = Φ(*A* × *D* × *C*), where *A* denotes the origin-licensing architecture, *D* the metabolic and kinase drives, *C* the chromatin context, and Φ the phase-control term governing cell-cycle permissiveness. The model predicts (i) all-or-none S-phase entry, (ii) synergistic inhibition when multiple pathways are partially reduced, and (iii) reversible arrest through checkpoint-mediated suppression of Φ. A simple mathematical formulation enables stability analysis and simulation using standard nonlinear control methods. The framework can be falsified by perturbation-matrix experiments measuring whether replication onset scales multiplicatively rather than additively with *A*, *D*, and *C*. By formalizing replication as a threshold-governed system, ARCH × Φ links molecular control of genome duplication with broader principles of biological decision-making, providing a quantitative bridge between cell-cycle dynamics and systems theory.

## 1 Introduction

The accurate duplication of genetic material is among the most fundamental and evolutionarily conserved events in biology. DNA replication does not occur continuously but initiates with precise spatial and temporal regulation, ensuring that each genome locus is copied once – and only once – during each cell cycle [1]. Since the elucidation of the double-helical structure of DNA by Franklin, Watson and Crick [2], biologists have sought to understand how cells determine *when* and *where* replication begins, and how this decision integrates molecular architecture, metabolic readiness, and chromatin context.

Early models, such as the replicon theory of Jacob, Brenner, and Cuzin [3], formalized the concept of discrete origin sites that license the initiation of replication. Subsequent decades of molecular dissection have described the proteins and checkpoints governing this process, yet a unifying theoretical framework remains lacking. Current descriptions are often modular – treating origin licensing, cyclin-dependent kinase activation, and chromatin accessibility as independent control layers – without a single formalism explaining their convergence.

**Figure 1:**
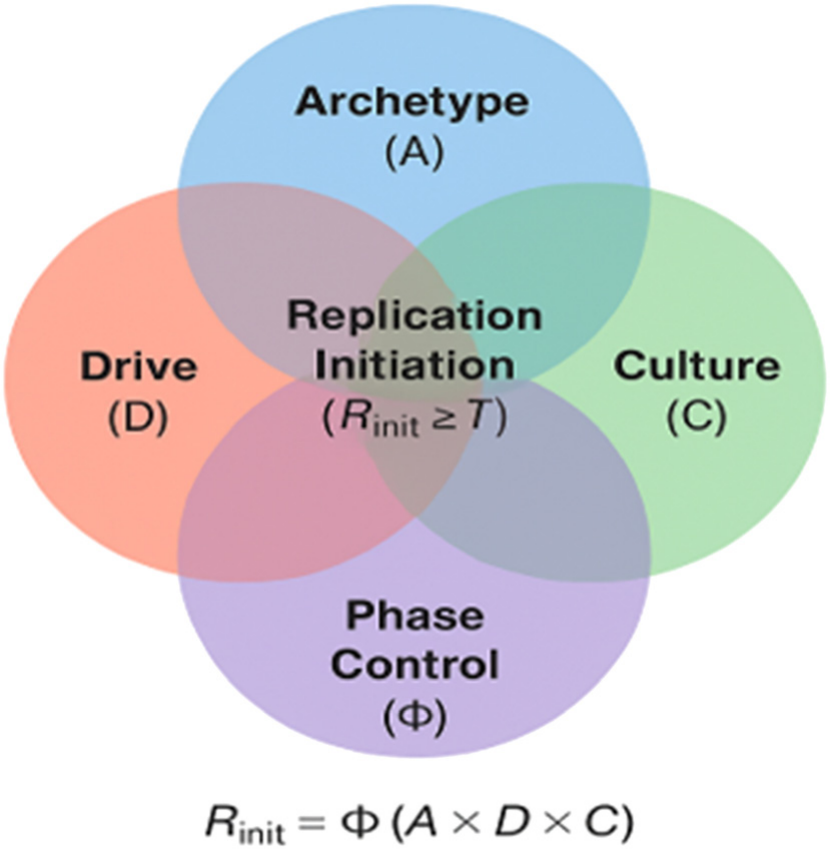
Conceptual structure of the ARCH × Φ model for replication initiation. Replication occurs only when four readiness domains converge above a critical threshold: *A* (Archetype) – structural and molecular licensing architecture (ORC, MCM, Cdc6); *D* (drive) – metabolic and kinase readiness (CDK activity, ATP/dNTP availability); *C* (Culture) – chromatin accessibility and 3D genome topology (H3K4me3-enriched, H3K9me3-repressed domains); Φ (phase control) – global cell-cycle permissiveness integrating checkpoints and *G*_1_/*S* transition status. Replication initiation occurs only when Φ(*A* × *D* × *C*) exceeds the threshold *T*, unifying architectural, energetic, contextual, and temporal determinants within a single quantitative framework.

Recent theoretical work has sought to represent cellular decisions as *threshold-governed* phenomena rather than linear sequences [4], 5]. Building on this perspective, the present study extends the ARCH framework, which has recently been formalized as a neuro-evolutionary model of behavioral execution [6]. The ARCH model posits that any organized biological action arises only when four essential conditions align above a critical threshold: Archetype, the structural architecture enabling the act; Drive, the energetic or motivational impetus; Culture, the contextual or environmental modulation; and Φ, a phase-control or homeostatic term representing baseline permissiveness. This framework is illustrated in Figure 1.

Here, this behavioral grammar is translated from higher-order systems to the molecular domain of DNA replication. The model presented herein hypothesizes that the initiation of genome duplication obeys the same conjunctive threshold logic: replication occurs only when structural (*A*), metabolic (*D*), chromatin (*C*), and phase-dependent (Φ) requirements converge above a minimal product value. This formulation models replication initiation as a multiplicative function of structural, metabolic, chromatin, and phase-readiness factors. The following sections formalize this model, define measurable correlates of each component, and outline its explanatory and predictive implications for replication timing, checkpoint control, and cellular variability.

## 2 Theoretical framework: the ARCH × Φ model

### 2.1 Conceptual basis

Replication initiation can be regarded as a decision-making process in which the cell integrates multiple forms of readiness before executing genome duplication. Analogous threshold behavior is well established in physiology and neuroscience [7], 8]. The ARCH × Φ framework applies this logic to replication, representing it as a *conditionally gated system* whose activation depends on the multiplicative conjunction of four control domains. The model extends the generalized ARCH principle [6], formulated initially to describe neural behavioral execution, to the cellular scale. In both cases, an act occurs only when preconditions in structure, energy, context, and global permissiveness align. This conceptual transfer provides a unifying language linking behavioral, cellular, and molecular decision systems.

### 2.2 Formal definition

Let *A*, *D*, *C*, and Φ denote normalized variables representing, respectively:

– *A* (Archetype): structural and molecular licensing architecture, including origin recognition complexes (ORC), minichromosome maintenance (MCM) helicases, and associated loading factors [9], 10].
– *D* (Drive): metabolic and signaling readiness – principally cyclin-dependent kinase activity, nucleotide availability, and energy state [11], 12].
– *C* (Culture): chromatin accessibility and nuclear topology defining the contextual permissiveness of origins [13], 14].
– Φ (Phase Control): global cell-cycle permissiveness integrating checkpoint status and phase readiness [15].

Replication initiation (denoted *R*_init_) occurs when: *R*_init_ = Φ(*A* × *D* × *C*) ≥ *T*, where *T* is a threshold constant representing the minimum combined readiness required for initiation. If any component approaches zero, the product is nullified – that is, Φ(*A* × *D* × *C*) falls below *T* – and replication initiation is categorically blocked. This conjunctive logic reproduces the empirically observed “all-or-none” behavior of S-phase entry [16]. A detailed derivation of this equation, its normalization procedure, and its stability properties are provided in Appendix A, which formalizes the mathematical behavior of the ARCH × Φ system.

### 2.3 Component definitions and correlates

#### 2.3.1 Archetype (*A*): replication architecture

A quantifies the presence and integrity of licensed origins and associated initiation machinery. Experimentally, *A* can be estimated by ORC/MCM occupancy, number of pre-replicative complexes, or origin competence assays [9]. Loss-of-function mutations in ORC or cell division cycle 6 (Cdc6) abolish origin firing regardless of signaling state [10].

#### 2.3.2 Drive (*D*): metabolic and signaling readiness

D captures the cell’s energy and cyclin-dependent kinase (CDK) activity. CDK2 and CDK4/6 activation, adenosine triphosphate (ATP) concentration, and deoxyribonucleotide triphosphate (dNTP) pool size contribute to *D* [11], 12]. When CDK activity or nucleotide supply is suppressed, initiation fails despite intact licensing.

#### 2.3.3 Culture (*C*): chromatin context

C denotes chromatin accessibility and nuclear organization. Euchromatic domains (H3K4me3-enriched) correspond to high *C*; heterochromatin or lamina-associated domains (H3K9me3), heterochromatin protein (HP1) correspond to low *C* [13], 14]. Chromatin remodeling that increases accessibility can elevate *C* and advance replication timing.

#### 2.3.4 Phase control (Φ): cell-cycle permissiveness

Φ represents global readiness determined by cell-cycle checkpoints. In *G*_0_ or mitosis, Φ ≈ 0; in late *G*_1_/*S*, Φ ≈ 1. DNA damage or checkpoint activation transiently reduces Φ by CDK inhibition [15], 17].

### 2.4 Threshold behavior and convergence

**Figure 2:**
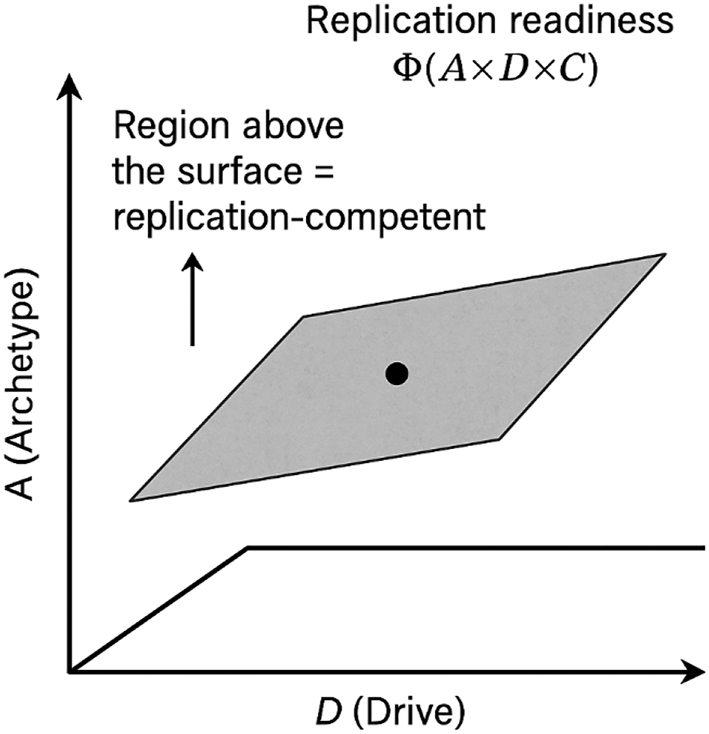
Conceptual threshold surface Φ(*A* × *D* × *C*) = *T* separating replication-competent (above) from non-competent (below) states. Increasing Φ (e.g., checkpoint release) lowers the surface, expanding the competent region; reducing Φ raises it.

In a hypothetical three-dimensional space with axes *A*, *D*, and *C*, the surface Φ(*A* × *D* × *C*) = *T* defines the boundary separating replication-competent from non-competent states [13], 14], 16]. This relationship is represented schematically in Figure 2. Increasing Φ (for example, after checkpoint release) lowers this threshold surface, whereas reducing Φ raises it. This schematic representation provides an intuitive map of replication readiness and predicts both synergistic and compensatory relationships among the contributing variables.

Because the ARCH × Φ formulation is multiplicative, the influence of each variable is strictly conjunctive: because the model is multiplicative, loss of any component prevents initiation. If any element – structural (*A*), metabolic (*D*), chromatin (*C*), or phase control (Φ) – is absent or falls below its critical value, the composite product Φ(*A* × *D* × *C*) drops beneath the threshold *T*, and initiation is categorically blocked (1 × 1 × 0 = 0).

At the molecular level, origin activation entails energy expenditure for both local DNA strand separation (“melting”) and elastic deformation of the surrounding chromatin [10], 13], 14]. These physical transitions define the immediate energetic barrier to initiation, such that the threshold *T* represents the combined cost of strand separation and bending, overcome only when Φ(*A* × *D* × *C*) provides sufficient energetic drive and structural alignment. In this view, chromatin accessibility (*C*) modulates both melting and bending energies: open euchromatin lowers the barrier, whereas compact heterochromatin raises it.

## 3 Model predictions and system behavior

### 3.1 Threshold-dependent initiation

The ARCH × Φ model predicts that replication initiation behaves as a threshold-governed, all-or-none event. Only when the combined product Φ(*A* × *D* × *C*) exceeds a defined threshold *T* does genome duplication commence. Because the system is multiplicative, increases in one variable cannot compensate for the absence of another; each domain is a necessary condition for initiation, and the product collapses when any term approaches zero. This conjunctive architecture mirrors experimental observations that origin firing requires simultaneous satisfaction of licensing, metabolic activation, chromatin accessibility, and a permissive cell-cycle phase [9], [10], [11], [12], [13], [14], [15]. Classical cell-fusion experiments showed that *G*_2_ nuclei exposed to S-phase cytoplasm do not re-initiate replication, despite abundant replication factors, because licensed origins are absent (*A* ≈ 0) [18]. Likewise, hydroxyurea-induced depletion of nucleotide pools or CDK inhibition prevents initiation even with licensed origins and accessible chromatin (*D* ≈ 0) [11], 19]. Heterochromatin compaction, while leaving *A* and *D* intact, suppresses firing of local origins (*C ≈ *0) [13]. Each of these conditions exemplifies the rule that replication ceases whenever any component of the multiplicative product approaches zero.

### 3.2 Synergistic interactions

The multiplicative form inherently encodes synergy among the four variables. Incremental changes in one component can yield nonlinear outcomes when others are near their thresholds. For example, moderate decreases in both *D* and *C* together can abolish replication even if neither alone is inhibitory – consistent with supra-additive effects observed during combined CDK inhibition and chromatin compaction [20], 21]. Graphically, this behavior corresponds to a steep, sigmoidal activation surface in *A*–*D*–*C* space. A deterministic contour plot of Φ(*A* × *D* × *C*) shows a narrow transition region separating quiescent and replicating states. Stochastic sampling of *A*, *D*, and *C* values (e.g., from beta distributions) generates bimodal populations – cells either fully initiate replication or remain inactive – reproducing the experimentally observed bistability of S-phase entry [16], 22].

### 3.3 Once-per-cycle replication

The model naturally explains the once-per-cycle rule of eukaryotic replication. During the S phase, licensed origins are consumed; consequently, *A* decreases as replication progresses. As licensed origins are consumed, further initiation ceases. In mitosis, high CDK1 activity suppresses licensing and reduces Φ to ≈0, enforcing a refractory period before relicensing [23]. As the cell exits mitosis, Φ gradually increases, reinstating competence in the next cycle. This interplay between declining A and oscillating Φ reproduces the observed periodicity in replication competence.

### 3.4 Checkpoint control and stress responses

Checkpoint signaling modulates replication by altering *D* and Φ. Activation of ATR or ATM in response to DNA damage inhibits CDK2 and stabilizes Cdc6, effectively lowering *D* and Φ below threshold [24]. Chromatin-based feedback further modulates *C*, for instance, by recruiting replication timing regulatory factor 1 (Rif1) and protein phosphatase 1 (PP1) complexes that locally suppress origin firing [25]. Such mechanisms map precisely onto the multiplicative structure of the model: checkpoint activation unaligns the required variables, reducing the overall product below *T* and halting initiation until repair is complete.

### 3.5 Early versus late S-phase dynamics

Replication timing domains arise from spatial and temporal heterogeneity in *C* and *A*. Early replicating euchromatic regions have high *C* and abundant licensed origins (*A* ≈ 1). In contrast, late-replicating heterochromatin exhibits lower *C* and fewer competent origins. Regions with higher *A* and *C* values cross the threshold earlier, leading to earlier replication [13], 14], 26]. Temporal modulation of Φ by checkpoint activity can further shift timing globally, explaining stress-induced delays in S-phase progression [24]. In this view, the spatially resolved replication program emerges not from a dedicated timing circuit but from distributed variations in *A*, *D*, and *C* within the same gating equation.

### 3.6 Population level variability

Because *A*, *D*, *C*, and Φ each fluctuate across single cells, the model predicts a broad yet quantized distribution of replication initiation states within populations. Single-cell analyses have shown discrete clusters of S-phase onset times rather than a continuum [27]. The ARCH × Φ formalism reproduces this pattern: stochastic variation around the threshold creates two dominant states – below-threshold (quiescent or *G*_1_) and above-threshold (S-phase entry). This bistability provides a mechanistic basis for population heterogeneity without invoking additional regulatory layers.

## 4 Comparative and theoretical context

### 4.1 Positioning within threshold and control theory

The ARCH × Φ model conceptualizes DNA replication initiation as a nonlinear threshold system governed by multiplicative conditionality. This formulation aligns with established control-theoretic principles of *state-dependent switching* and *threshold-crossing dynamics*, in which complex outputs arise only when multiple control variables exceed critical limits [20], 28]. In such systems, equilibrium stability changes abruptly once a control manifold is crossed – a behavior described mathematically as a bifurcation or switching transition [28], 29].

Where traditional molecular models depict replication as a sequence of reactions, ARCH × Φ treats it as a stability problem: replication initiation corresponds to a transition from a non-replicating to a replicating steady state when the product Φ(*A* × *D* × *C*) surpasses *T*. The multiplicative form imposes conjunctive logic analogous to multi-input control gates in dynamical systems [30]. This framing permits formal stability analysis and simulation using established nonlinear-systems approaches, while remaining faithful to empirical cell-cycle biology.

### 4.2 Relation to bistable cell-cycle switches

Classical models of the *G*_1_/*S* transition attribute bistability to feedback between retinoblastoma (Rb) and E2F transcription factors – the Rb–E2F switch [16], 31], 32]. These feedback loops generate hysteresis and irreversible S-phase commitment once CDK activity surpasses a critical threshold. The ARCH × Φ model generalizes this principle: bistability arises not solely from feedback but from multiplicative gating of required inputs. When *A* and *C* ≈ 1 and Φ acts as a binary variable, ARCH × Φ collapses to the Rb–E2F switch; when *A* and *C* vary continuously, the system defines a multidimensional threshold surface rather than a single critical point. Thus, the model embeds molecular feedback within a broader conjunctive framework that also incorporates architectural and chromatin constraints.

### 4.3 Relation to stochastic and probabilistic origin-firing models

Existing stochastic models describe origin firing as probabilistic, determined by local origin competence and external modulation [33], 34]. Although these frameworks reproduce genome-wide replication-timing distributions, they assume that variables contribute additively or independently. In contrast, ARCH × Φ introduces conditional multiplicativity: an origin fires only when Φ(*A* × *D* × *C*)_*i* ≥ *T*_*i*. This logic explains the supra-additive inhibition observed when multiple pathways are partially suppressed [21], 35] and provides a mechanistic basis for population-level bistability in single-cell replication timing [27], 36]. In dynamical-systems terms, variations in *A*, *D*, and *C* displace a cell’s state relative to the threshold manifold, and stochastic sampling around this boundary yields the experimentally observed coexistence of early- and late-replicating populations.

### 4.4 Integration with additive and energy-based frameworks

Additive models approximate replication timing as the sum of chromatin state, origin density, and replication-factor abundance [37]. Although statistically accurate, additive combinations yield linear scaling and cannot produce discontinuous transitions. In contrast, the multiplicative gate in ARCH × Φ introduces nonlinear amplification and saturation – features characteristic of biological switches. Energetically, this corresponds to requiring that the system’s total activation potential, Φ(*A* × *D* × *C*), exceed a threshold, *T* to initiate replication. Thus, ARCH × Φ provides a biophysically interpretable bridge between empirical regression frameworks and nonlinear control theory.

### 4.5 Conceptual continuity with the general ARCH framework

The ARCH × Φ model extends the generalized ARCH principle of biological behavior [6], wherein organized actions occur only when architecture, drive, context, and phase align above threshold. Applied to the molecular domain, this same logic governs the cellular decision to replicate. Genome duplication thus fits within a hierarchy of conditionally gated behaviors – neural, cellular, and molecular – described by a shared mathematical grammar. Incorporating control-theoretic stability concepts [28], 29] formalizes this hierarchy: across scales, the shift from inactivity to execution corresponds to crossing a critical manifold in multidimensional parameter space.

## 5 Applications and case scenarios

### 5.1 Application of the model to cellular contexts

The ARCH × Φ framework provides a quantitative grammar for diverse replication behaviors across cell types. Parameterizing the model for each component – Archetype, Drive, Culture, and Phase – allows physiological and perturbed states to be represented with measurable correlates [9], [10], [11], [12], [13], [14], [15]. In rapidly dividing embryonic or stem cells, *A*, *D*, and *C* remain near maximal, and Φ is constitutively high due to attenuated checkpoints [26], 38]; the system therefore stays above threshold, supporting continuous or shortened cell cycles. In contrast, quiescent or differentiated cells show reduced *D* and Φ. Although origins remain licensed (*A* ≈ 1), replication is suppressed because overall readiness falls below threshold. This distinction explains why replication reactivation in differentiated cells requires metabolic and checkpoint reprogramming.

### 5.2 DNA-damage and checkpoint inhibition scenarios

During genotoxic stress, ataxia telangiectasia and Rad3-related (ATR) and ataxia telangiectasia mutated (ATM) kinase signaling suppresses cyclin-dependent kinase 2 (CDK2) activity and lowers Φ [24], 25], shifting the system below the initiation threshold even when *A* and *C* remain intact. This explains the rapid replication arrest in hydroxyurea-treated or UV-irradiated cells [19], 24]. Recovery of checkpoint control restores CDK activity and raises Φ, allowing replication to resume. Thus, transient stress drives reversible crossing of the threshold manifold: replication halts globally yet reversibly because checkpoint regulation modulates Φ rather than dismantling origin architecture.

### 5.3 Early-versus late-S-phase domains

Spatial variation in *A* and *C* underlies the biphasic pattern of S-phase replication [13], 14], 26]. Euchromatic regions (*C* ≥ 0.7) with dense licensed origins (*A* ≈ 1) reach the threshold first and replicate early, whereas heterochromatin (*C* ≤ 0.3, *A* < 0.5) requires stronger Drive and Phase permissiveness to fire later. Checkpoint-mediated reductions in Φ shift this timing curve globally, delaying both domains. Replication timing, therefore, emerges as a distributed property of the same multiplicative gate rather than from a dedicated temporal controller.

### 5.4 Bulk-population versus single-cell predictions

In multicellular contexts, intercellular signaling and proximity add a layer of modulation: neighboring cells can influence each other’s metabolic drive, chromatin state, and checkpoint control. This coupling extends the ARCH × Φ framework from single-cell dynamics to collective behavior, allowing replication readiness to propagate across populations. Because *A*, *D*, *C*, and Φ fluctuate among cells, the model predicts heterogeneity even in genetically identical cultures. Stochastic variation in these components determines whether an individual cell surpasses the replication threshold, generating bimodal – replicating or quiescent – states consistent with single-cell Repli-Seq data [27], 36]. At the bulk level, ensemble averaging of these binary states produces apparent gradients in replication probability, reconciling population and single-cell observations without invoking separate regulatory programs.

### 5.5 Model-based experimental design

The framework suggests direct perturbation experiments for empirical validation. A three-factor design varying origin licensing, kinase activity, and chromatin accessibility can map the predicted threshold surface:

(1) *A* modulation – partial ORC or MCM depletion;
(2) *D* modulation – CDK2 inhibition or nucleotide depletion;
(3) *C* modulation – histone-deacetylase inhibition or heterochromatin tethering.

Replication output measured by bromodeoxyuridine (BrdU) incorporation or single-cell sequencing should display multiplicative – not additive – suppression. Combined partial perturbations (e.g., 50 % reduction in *D* plus 50 % reduction in *C*) are expected to produce supra-additive inhibition, providing a clear test of the model’s conjunctive logic.

### 5.6 Translational implications

Many cancer cells exhibit chronically high *D* from oncogenic signaling and impaired checkpoint control, yielding Φ ≈ 1 but defective *A* and *C* due to licensing stress and chromatin instability [39], 40]. The ARCH × Φ framework interprets this as a misaligned state in which excessive *D* and Φ sustain replication above threshold despite structural and chromatin deficits, promoting genomic instability. Therapeutic strategies that rebalance these parameters – such as concurrent CDK inhibition (reducing *D*) and chromatin-stabilizing agents (restoring *C*) – could selectively reduce tumor cell replication competence while sparing normal tissues. This reasoning quantitatively explains the synergy of combined cell-cycle and epigenetic interventions [41] and links the model’s parameters to clinically testable targets summarized in Appendix B.

## 6 Limitations and future directions

### 6.1 Conceptual simplifications

The ARCH × Φ model represents replication initiation as a threshold-governed system, reducing hundreds of biochemical reactions to four composite control variables. This abstraction clarifies the governing logic but omits the detailed feedback and post-translational regulation operating within each domain [9], [10], [11], [12, 23]. *In vivo*, *A*, *D*, *C*, and Φ are interdependent: metabolic signaling affects chromatin accessibility, and checkpoint activation modifies both licensing and drive. The framework should therefore be viewed as a first-order conditional scaffold for organizing these interrelations rather than a full mechanistic map.

### 6.2 Quantitative parameterization

Implementing the model requires empirical scaling of each variable (0–1) and estimation of the threshold *T*. *A* can be quantified by genome-wide ORC/MCM occupancy or licensing-factor abundance [9], 10]; *D* by CDK activity assays and nucleotide-pool measurements [11], 12]; *C* by ATAC-seq or histone-mark profiling [13], 14]; and Φ by checkpoint or cyclin-phosphorylation readouts [15], 24]. Defining *T* involves correlating these normalized metrics with replication onset at single-cell resolution, potentially using logistic regression or control-system identification methods [28], 29]. Until such calibration is achieved, predictions remain qualitative, but the model’s structure offers a practical roadmap for parameter extraction.

### 6.3 Integration with stochastic and dynamical frameworks

Future extensions should express the model as a time-dependent system, d*R*/d*t* = *f*(Φ, *A*, *D*, *C*) − *g*(*R*), where *f* represents activation and *g* denotes inhibitory feedback. This formulation enables the simulation of replication kinetics and the stability analysis using established nonlinear and delay-differential methods [28], [29], [30]. Adding stochastic noise terms could capture the heterogeneity observed in origin firing and S-phase duration [33], 34], 36], thereby linking the conceptual and quantitative domains for direct comparison with single-cell data.

### 6.4 Multiscale extensions

The same governing logic can be applied hierarchically. At the sub-nuclear scale, each origin has local *A*, *D*, *C*, and Φ values; at the cellular level, these aggregate into a replication probability; and across tissues, the averaged Φ reflects the developmental or pathological state. Embedding ARCH × Φ within agent-based or network models would allow exploration of how replication-timing domains arise from local interactions. This multiscale view aligns with systems-biology efforts to integrate chromatin architecture, metabolism, and cell-cycle control within unified dynamic frameworks [5], 32], 42].

### 6.5 Experimental roadmap

Empirical testing may proceed in three stages:

1. Perturbation matrix experiments: systematically vary *A* (MCM or ORC knockdown), *D* (CDK or nucleotide modulation), and *C* (chromatin accessibility) while monitoring replication entry [19], 21]; fit initiation frequencies to the predicted multiplicative model.
2. Single-cell correlation analysis: quantify each parameter by live-cell reporters or multiplexed imaging and regress replication onset against Φ(*A* × *D* × *C*); a sigmoidal relationship would confirm the threshold hypothesis.
3. Dynamic control testing: use optogenetic or chemical tools to modulate Φ transiently and demonstrate reversible threshold crossing in real time [24], 38].

These experiments would transform the model from a qualitative synthesis into a quantitatively testable framework.

### 6.6 Broader theoretical implications

The gating architecture described here may extend beyond replication to other conditionally executed cellular programs – such as transcriptional activation, immune signaling, or developmental fate transitions [5], 39], 42]. In each, structural readiness, metabolic drive, contextual modulation, and phase alignment act conjunctively to determine execution. Integrating these processes within the same ARCH grammar could yield a scalable framework for biological decision-making across molecular, cellular, and behavioral levels.

## 7 Discussion and broader implications

### 7.1 Conceptual integration

The ARCH × Φ model reframes DNA-replication initiation as a conditionally gated event rather than a linear biochemical cascade. It unifies structural licensing, metabolic drive, chromatin context, and phase control within one quantitative framework, explaining both complete inhibition when any prerequisite is missing and synergistic suppression under partial perturbation [11], 20], 21]. The model formalizes the long-recognized all-or-none nature of S-phase entry within a mathematical architecture consistent with control theory and systems biology principles [28], [29], [30], linking empirical molecular biology to the general logic of nonlinear systems analysis.

### 7.2 Relationship to existing paradigms

Classical models, such as the Rb–E2F feedback switch, describe *G*_1_/*S* commitment as a bistable transition driven by reciprocal activation and inhibition [16], 31], 32]. ARCH × Φ generalizes this behavior by embedding bistability within a broader conjunctive manifold: replication begins only when readiness across all four domains exceeds threshold. Unlike additive or feedback-only schemes, this framework captures conditional necessity – each control axis is indispensable – thereby integrating feedback kinetics, probabilistic origin firing, and structural licensing within a unified grammar of control.

### 7.3 Evolutionary and cross-scale continuity

The progressive coupling of *A*, *D*, *C*, and Φ likely reflects an evolutionary deepening of control – from prebiotic replication governed by passive energetic constraints to eukaryotic systems in which initiation became an information-driven decision. The ARCH × Φ framework thus outlines a conserved decision grammar elaborated rather than replaced through evolution.

Replication control exemplifies a universal principle: complex acts occur only when multiple constraints align. The same logic governs behavior at higher levels of organization – from neural reflexes to multicellular coordination. Extending the original ARCH behavioral model [6] to the molecular scale reveals a shared computational architecture for conditional activation; replication initiation becomes the cellular analogue of behavioral execution, both rooted in structural readiness, energetic drive, contextual modulation, and systemic control. Energetic optimization likely reinforced this architecture. Primitive systems replicated whenever conditions coincided, regardless of cost, whereas modern cells integrate energetic expense into the decision, initiating only when investment yields functional benefit. This shift from permissive to efficient convergence represents a further evolutionary refinement of the ARCH × Φ logic.

### 7.4 Theoretical implications for systems biology

Framing replication as a gated nonlinear system embeds it within the broader landscape of biological control. Such representation enables stability analysis, bifurcation mapping, and predictive simulation long used in engineering but underapplied in cell biology [28], 29]. Replication exemplifies a bistable switch: the system remains non-replicating until the control surface is crossed, after which it transitions to a stable replicating state. This dual-state topology parallels phase transitions in physics and decision thresholds in neural circuits [7], 8], 16], providing a quantitative bridge between molecular regulation and nonlinear dynamics.

### 7.5 Implications for disease, therapeutics, and synthetic biology

Pathological and engineered states can be viewed as distortions – or deliberate manipulations – of the ARCH × Φ balance. In cancer, oncogenic signaling elevates *D* and Φ while chromatin dysregulation or licensing defects reduce *A* and *C*; replication persists above threshold, producing genomic instability [39], 40]. Therapeutic strategies that jointly attenuate *D* (CDK inhibition) and restore *C* (chromatin-stabilizing or checkpoint-restoring agents) could lower overall readiness and suppress tumor proliferation. Conversely, synthetic or minimal cells deliberately maintain high *D* and permissive Φ under simplified constraints to accelerate replication. These contrasting regimes illustrate the model’s predictive reach and its utility for designing multi-target interventions or controllable self-replicating systems, as summarized in Appendix B.

### 7.6 Generalization beyond DNA replication

Threshold-governed dynamics likely extend to other organellar and cellular programs. For instance, mitochondrial biogenesis requires the alignment of structural, energetic, contextual, and phase factors: *A* (the mitochondrial apparatus, including mitochondrial DNA and transcription factor *A*, mitochondrial – TFAM), *D* (energetic and signaling inputs such as the adenosine monophosphate/adenosine triphosphate ratio and activation of peroxisome proliferator-activated receptor gamma coactivator 1-alpha – PGC-1α), *C* (nuclear–mitochondrial coordination, proteostasis, and membrane dynamics), and Φ (global permissivity, reflecting cell-cycle phase or stress checkpoints). Disruption of any component – such as TFAM deletion, suppression of AMP-activated protein kinase (AMPK), or chromatin closure of oxidative phosphorylation (OXPHOS) genes – abolishes biogenesis despite other terms remaining intact. This mirrors the “zero-term veto” observed in apoptosis, where loss of the pro-apoptotic B-cell lymphoma 2 (Bcl-2) family proteins Bcl-2-associated X protein (Bax) and Bcl-2 antagonist/killer 1 (Bak) prevents mitochondrial outer membrane permeabilization even under maximal pro-death drive. Similar conjunctive logic may govern transcriptional bursts, immune activation, differentiation, and behavioral outputs [5], [42], [43], [44]. Recognizing this shared control grammar could yield a unified, scale-invariant theory of biological execution, consistent with the view that life organizes information through hierarchical, threshold-based systems rather than linear causal chains.

### 7.7 Summary of theoretical perspective

The ARCH × Φ model provides a concise yet comprehensive framework for replication initiation, consolidating diverse empirical findings into a single governing equation. It explains bistability and synergistic inhibition without invoking complex feedback circuits and situates replication within a universal theory of biological behavior. Because its predictions are experimentally falsifiable through multifactor perturbations of *A*, *D*, *C*, and Φ, the model offers a tractable basis for quantitative testing and for extending control-theoretic analysis across biological scales – from genome duplication to cognition.

## 8 Conclusions

The ARCH × Φ model reframes DNA-replication initiation as a conditionally gated, threshold-driven decision rather than a linear biochemical sequence. Replication commences only when architectural integrity (*A*), metabolic drive (*D*), chromatin context (*C*), and phase control (Φ) align above a critical readiness threshold. This framework unites structural, metabolic, and regulatory determinants within a single quantitative expression, accounting for bistability, synergistic inhibition, and once-per-cycle fidelity without requiring complex feedback networks. Extending the validated ARCH behavioral framework from neural systems to molecular replication exposes a common decision logic operating across biological scales. The model transforms replication from a descriptive molecular process into a falsifiable, predictive system – one that links cellular control to general principles of nonlinear dynamics and decision theory. Its implications extend beyond genomics, offering a quantitative language for understanding dysregulated replication in cancer, designing multi-target therapeutics, and engineering controllable self-replicating systems. In essence, ARCH × Φ defines replication as a universal decision principle of living matter, where structure, energy, context, and control must converge for life to act.

## Appendix A: Mathematical formulation and normalization

### A.1 Model definition

Replication initiation is represented as a threshold-governed multiplicative system:

$$R\left(t\right)=\Phi \left(t\right)\times \left[A\left(t\right)\times D\left(t\right)\times C\left(t\right)\right]\text{,}$$

where *A*(*t*), *D*(*t*), *C*(*t*), and Φ(*t*) ∈ [0,1] are normalized state variables that vary continuously in time. Replication proceeds when *R*(*t*) ≥ *T*, with *T* denoting the critical threshold for activation. Each variable represents a distinct control domain:

Appendix Table: Model Variable Definitions.

**Table:**

Variable	Definition	Typical empirical range
*A*	Origin licensing and molecular architecture	0–1; approaches 1 in late *G*_1_
*D*	Metabolic/kinase drive	0–1; threshold ≈0.6
*C*	Chromatin/contextual accessibility	0–1; domain-dependent
Φ	Global phase-control readiness	0–1; ≈1 in late *G*_1_/*S*

### A.2 Time-dependent formulation

To analyze dynamical properties, the steady-state relation can be extended to a differential form:

$$dR/dt=k1\times \Phi \left(t\right)\times \left[A\left(t\right)D\left(t\right)C\left(t\right)\right]-k2\times R\left(t\right)\text{,}$$

where *k*1 is the activation rate constant and *k*2 represents deactivation or inhibitory decay (e.g., checkpoint enforcement). At equilibrium: *R** = (*k*1/*k*2) × Φ(*A D C*). Replication is stable and sustained only when *R** ≥ *T*. Linearization around *R** yields the local stability condition: d(d*R*/d*t*)/d*R*|*R** = −*k*2 < 0, indicating that once initiated, the system relaxes toward a stable steady state until one or more variables decrease below threshold.

### A.3 Threshold surface and stability

The critical surface separating replicative from non-replicative states is defined by Φ(*A D C*) = *T*. For Φ fixed, this surface partitions the three-dimensional *A*–*D*–*C* space into stable (*R* > *T*) and inactive (*R* < *T*) domains. Differentiation of the activation function gives:

∂R/∂A=ΦDC,∂R/∂D=ΦAC,∂R/∂C=ΦAD

This demonstrates mutual dependence: a decrement in any variable proportionally scales the effect of the others. Such multiplicative coupling produces nonlinear amplification and threshold phenomena characteristic of bistable biological switches.

### A.4 Normalization procedure

Each variable may be normalized to its physiological range:

$$X_{\text{norm}\left(t\right)}=\left[X\left(t\right)-X_{\min}\right]/\left[X_{\max}-X_{\min}\right],X\in \left\{A,D,C,\Phi \right\}$$

Normalization ensures comparability across experimental conditions and enables estimation of T as the point at which the measured replication probability transitions from 0 to 1. Single-cell data can be fitted to: *P*(replication) = 1/[1 + exp(−*β*[Φ(*A D C*) − *T*])], yielding *β* as the slope (steepness) of the activation curve.

### A.5 Relation to stability theory

The threshold-crossing property conforms to the general structure of state-dependent switching systems described in nonlinear control theory. In such systems, stability is governed by Lyapunov functions whose derivatives change sign upon crossing the manifold Φ(*A D C*) = *T*. When *R*(*t*) < *T*, the system remains in the quiescent attractor; when *R*(*t*) > *T*, trajectories converge to the active attractor. This dual-state topology provides a formal justification for the empirical bistability of S-phase commitment. Hence, ARCH × Φ can be interpreted as a biological realization of a nonlinear control system, where architectural, metabolic, and chromatin terms act as multiplicative control inputs and Φ as a global gain or switching coefficient.

### A.6 Implications for simulation

This formulation enables computational implementation using standard nonlinear-systems solvers. Sampling *A*, *D*, *C*, and Φ from empirical distributions and integrating the time-dependent equation reproduces population-level replication dynamics, bistability, and recovery after perturbation. These simulations can be compared with experimental datasets (e.g., single-cell Repli-Seq, chromatin-state maps) to infer parameter distributions and validate the theoretical threshold, *T*. References [28], [29], [30] correspond to established literature on nonlinear control and stability analysis. These predicted outcomes serve as testable benchmarks for future experiments, particularly in single-cell replication timing and checkpoint modulation studies. Each scenario provides a falsifiable prediction regarding the quantitative relationship among *A*, *D*, *C*, and Φ.

## Appendix B

Key perturbation predictions derived from the ARCH × Φ framework. The table highlights expected replication outcomes and theoretical interpretations.

**Table:**

Condition	Variable Configuration (*A*, *D*, *C*, Φ)	Predicted product Φ(*A* × *D* × *C*)	Expected outcome	Experimental analog/validation	Interpretation
Normal proliferating cell	*A* ≈ 1, *D* ≈ 1, *C* ≈ 1, Φ ≈ 1	≥*T*	Robust replication initiation (normal S phase).	Standard cycling fibroblast or stem cell cultures.	All control axes aligned above threshold → active replication.
Quiescent cell (*G*_0_)	*A* ≈ 1, *D* ≈ 0.2, *C* ≈ 0.6, Φ ≈ 0	<*T*	Replication silent.	Contact-inhibited or serum-starved fibroblasts.	Low drive and Φ prevent activation despite intact licensing.
Checkpoint-activated cell	*A* ≈ 1, *D* ≈ 0.4, *C* ≈ 0.6, Φ ≈ 0.3	<*T*	Replication arrests until repair is complete.	Hydroxyurea, ATR/ATM activation assays.	Checkpoint signaling suppresses Φ and *D* below threshold.
Chromatin-compacted domain	*A* ≈ 1, *D* ≈ 1, *C* ≈ 0.2, Φ ≈ 1	<*T* locally	Late or suppressed origin firing.	Heterochromatin regions; lamina-associated domains.	Low chromatin accessibility reduces local *C* and delays initiation.
Embryonic/oncogenic cell	*A* ≈ 0.8, *D* ≈ 1.2, *C* ≈ 1, Φ ≈ 1.2	≫*T*	Accelerated or unscheduled replication.	Embryonic stem cells; tumor cells with high CDK and relaxed checkpoints.	Excessive drive and Φ overcome partial structural deficits → replication stress.
Partial perturbations (*A*↓, *D*↓)	*A* = 0.5, *D* = 0.5, *C* = 1, Φ = 1	=0.25 < *T*	Replication fails despite moderate depletion.	Dual inhibition: ORC depletion + CDK inhibitor.	Multiplicative synergy → supra-additive suppression of replication.
Checkpoint recovery	*A* ≈ 1, *D*↑, *C* ≈ 0.6, Φ↑	Crosses *T*	Replication restarts post-stress.	Release from hydroxyurea block or DNA-damage recovery.	Increasing *D* and Φ re-align the system above the threshold.

Abbreviations for Appendix B table: *A*, archetype; *D*, drive; *C*, culture (chromatin/contextual accessibility); *Φ*, chase control; *T*, threshold constant for initiation; ↑/↓, increase or decrease relative to normal values; ≫*T*, product greatly exceeds threshold; *<T*, product below threshold. ORC, origin recognition complex; MCM, minichromosome maintenance helicase; CDK, cyclin-dependent kinase; ATR/ATM, ataxia telangiectasia and Rad3-related/ataxia telangiectasia mutated kinases; BrdU, bromodeoxyuridine.

## References

[1] Méndez J, Stillman B (2003). Perpetuating the double helix: molecular machines at eukaryotic DNA replication origins. Bioessays.

[2] Watson JD, Crick FHC (1953). Molecular structure of nucleic acids: a structure for deoxyribose nucleic acid. Nature.

[3] Jacob F, Brenner S, Cuzin F (1963). On the regulation of DNA replication in bacteria. Cold Spring Harbor Symp Quant Biol.

[4] Ferrell JE (2012). Bistability, bifurcations, and Waddington’s epigenetic landscape. Curr Biol.

[5] Bertoli C, Skotheim JM, de Bruin R (2013). Control of cell cycle transcription during G1 and S phases. Nat Rev Mol Cell Biol.

[6] Rahman T, Zorumski CF, Meloy JR (2025). The ARCH model: a neuroevolutionary framework for behavioral execution. Front Psychiatry.

[7] Yao G, Lee TJ, Mori S, Nevins JR, You L (2008). A bistable Rb–E2F switch underlies the restriction point. Nat Cell Biol.

[8] Ferrell JE (2002). Self-perpetuating states in signal transduction: positive feedback, double-negative feedback and bistability. Curr Opin Cell Biol.

[9] Bell SP, Dutta A (2002). DNA replication in eukaryotic cells. Annu Rev Biochem.

[10] Bell SP, Stillman B (1992). ATP-dependent recognition of eukaryotic origins of DNA replication by a multiprotein complex. Nature.

[11] Sherr CJ, Roberts JM (1999). CDK inhibitors: positive and negative regulators of G1-phase progression. Genes Dev.

[12] Aird KM, Zhang R (2015). Nucleotide metabolism, oncogene-induced senescence and cancer. Cancer Lett.

[13] Bickmore WA, van Steensel B (2013). Genome architecture: domain organization of interphase chromosomes. Cell.

[14] Pope BD, Ryba T, Dileep V, Yue F, Wu W, Denas O (2014). Topologically associating domains are stable units of replication-timing regulation. Nature.

[15] Spencer SL, Cappell SD, Tsai FC, Overton KW, Wang CL, Meyer T (2013). The proliferation-quiescence decision is controlled by a bifurcation in CDK2 activity at mitotic exit. Cell.

[16] Konagaya Y, Rosenthal D, Ratnayeke N, Fan Y, Meyer T (2024). An intermediate Rb–E2F activity state safeguards proliferation commitment. Nature.

[17] Shaltiel IA, Krenning L, Bruinsma W, Medema RH (2015). The same, only different—DNA damage checkpoints and their reversal throughout the cell cycle. J Cell Sci.

[18] Rao PN, Johnson RT (1970). Mammalian cell fusion: studies on the regulation of DNA synthesis and mitosis. Nature.

[19] Santocanale C, Diffley JFX (1998). A Mec1- and Rad53-dependent checkpoint controls late-firing origins of DNA replication. Nature.

[20] Ferrell JE, Ha SH (2014). Ultrasensitivity part II: multisite phosphorylation, stoichiometric inhibitors, and positive feedback. Trends Biochem Sci.

[21] Hiraga SI, Ly T, Garzón J, Hořejší Z, Ohkubo YN, Endo A (2017). Human RIF1 and protein phosphatase 1 stimulate origin licensing but suppress origin activation. EMBO Rep.

[22] Rhind N, Yang SCH, Bechhoefer J (2010). Reconciling stochastic origin firing with defined replication timing. Chromosome Res.

[23] Blow JJ, Laskey RA (1988). A role for the nuclear envelope in controlling DNA replication within the cell cycle. Nature.

[24] Joo YK, Ramirez C, Kabeche L (2024). A TRilogy of ATR’s non-canonical roles throughout the cell cycle and its relation to cancer. Cancers.

[25] Mattarocci S, Shyian M, Lemmens L, Damay P, Altintas DM, Shi T (2014). Rif1 controls DNA replication timing in yeast through the PP1 phosphatase Glc7. Cell Rep.

[26] Nakatani T, Schauer T, Altamirano-Pacheco L, Klein KN, Ettinger A, Pal M (2024). Emergence of replication timing during early mammalian development. Nature.

[27] Bartlett DA, Dileep V, Baslan T, Gilbert DM (2022). Mapping replication timing in single mammalian cells. Curr Protoc.

[28] Michiels W, Niculescu SI (2007). Stability and stabilization of time-delay systems: an eigenvalue-based approach.

[29] Liberzon D (2003). Switching in systems and control.

[30] Sontag ED (2007). Monotone and near-monotone biochemical networks. Syst Synth Biol.

[31] Tyson JJ, Novak B (2001). Regulation of the eukaryotic cell cycle: molecular antagonism, hysteresis, and irreversible transitions. J Theor Biol.

[32] Novak B, Tyson JJ (1993). Numerical analysis of a comprehensive model of M-phase control in xenopus oocyte extracts and intact embryos. J Cell Sci.

[33] Gindin Y, Valenzuela MS, Aladjem MI, Meltzer PS, Bilke S (2014). A chromatin structure–based model accurately predicts DNA replication timing in human cells. Mol Syst Biol.

[34] Gilbert DM, Takebayashi SI, Ryba T, Lu J, Pope BD, Wilson KA (2010). Space and time in the nucleus developmental control of replication timing and chromosome architecture. Cold Spring Harbor Symp Quant Biol.

[35] Mansisidor AR, Risca VI (2022). Chromatin accessibility: methods, mechanisms, and biological insights. Nucleus.

[36] Vouzas AE, Gilbert DM (2023). Replication timing and transcriptional control: beyond cause and effect—part IV. Curr Opin Genet Dev.

[37] Chen Y, Liang R, Li Y, Jiang L, Ma D, Luo Q (2024). Chromatin accessibility: biological functions, molecular mechanisms and therapeutic application. Signal Transduction Targeted Ther.

[38] Blythe SA, Wieschaus EF (2015). Coordinating cell cycle remodeling with transcriptional activation at the drosophila MBT. Curr Top Dev Biol.

[39] Mathews CK (2015). Deoxyribonucleotide metabolism, mutagenesis and cancer. Nat Rev Cancer.

[40] Hills SA, Diffley JFX (2014). DNA replication and oncogene-induced replicative stress. Curr Biol.

[41] Scarpulla RC (2008). Transcriptional paradigms in mammalian mitochondrial biogenesis and function. Physiol Rev.

[42] Chakraborty D, Rengaswamy R, Raman K (2022). Designing biological circuits: from principles to applications. ACS Synth Biol.

[43] Tait SWG, Green DR (2010). Mitochondria and cell death: outer membrane permeabilization and beyond. Nat Rev Mol Cell Biol.

[44] Petermann E, Helleday T (2010). Pathways of mammalian replication fork restart. Nat Rev Mol Cell Biol.

